# An impact of age on respiratory syncytial virus infection in air-liquid-interface culture bronchial epithelium

**DOI:** 10.3389/fmed.2023.1144050

**Published:** 2023-03-14

**Authors:** Kazuhiro Ito, Leah Daly, Matthew Coates

**Affiliations:** National Heart and Lung Institute, Imperial College, London, United Kingdom

**Keywords:** age, respiratory syncytial virus, air-liquid-interface culture, CXCL8, IL-6, RANTES (regulated upon activation), mucin, dsDNA

## Abstract

**Background:**

Elderly people are known to be vulnerable to virus infection. However, this has not been appropriately tested in *in vitro* studies due to a lack of appropriate virus infection models. In this report, we investigated the impact of age on respiratory syncytial virus (RSV) in pseudostratified air-liquid-interface (ALI) culture bronchial epithelium, which more closely mimic human airway epithelium morphologically and physiologically, than submerged cancer cell line cultures.

**Methods:**

RSV A2 was inoculated apically to the bronchial epithelium obtained from 8 donors with different ages (28–72 years old), and time-profiles of viral load and inflammatory cytokines were analyzed.

**Results:**

RSV A2 replicated well in ALI-culture bronchial epithelium. The viral peak day and peak viral load were similar between donors at ≤60 years old (*n* = 4) and  > 65 years old (*n* = 4; elderly group), but virus clearance was impaired in the elderly group. Furthermore, area under the curve (AUC) analysis, calculated from viral load peak to the end of sample collection (from Day 3 to 10 post inoculation), revealed statistically higher live viral load (PFU assay) and viral genome copies (PCR assay) in the elderly group, and a positive correlation between viral load and age was observed. In addition, the AUCs of RANTES, LDH, and dsDNA (cell damage marker) were statistically higher in the elderly group, and the elderly group showed a trend of higher AUC of CXCL8, CXCL10 and mucin production. The gene expression of p21^CDKN1A^ (cellular senescence marker) at baseline was also higher in the elderly group, and there was a good positive correlation between basal p21 expression and viral load or RANTES (AUC).

**Conclusion:**

Age was found to be a key factor affecting viral kinetics and biomarkers post virus infection in an ALI-culture model. Currently, novel or innovative *in vitro* cell models are introduced for virus research, but when virus studies are conducted, similarly to working with other clinical samples, the age balance is important to obtain more accurate results.

## Introduction

Historically, virus infection research was conducted using submerged cancer cell line monolayer cultures (2D), which are susceptible to specific virus species. For example, MDCK cells were used for influenza, HEp-2 cells were used for respiratory syncytial virus (RSV), and HeLa cells were used for human rhinovirus (HRV). However, especially during the COVID-19 pandemic, we realized that the outcomes from cancer cell line research were not often translational to the clinic, as those are limited to a single cell type originally from a single donor (or sometimes from animals) having defect of host defense at molecular level, and submerged, thus, do not mimic 3D human tissue architecture and lack biochemical and biomechanical cues. These models are low cost and suitable for high throughput systems, but consequently, routinely require *in vivo* animal model validation. However, animal models are expensive, rarely accurately mimic human biological responses due to obvious differences in physiology, pathology and other genetic factors, as well as having ethical issues. Air-liquid interface (ALI)-culture airway epithelium are being increasingly recognized for their ability to overcome many of the disadvantages of submerged cell culture models (1–3). They consist of pseudostratified fully differentiated cells, cultured in transwell inserts, wherein the apical cells are exposed to the air and the basolateral cells fed by culture medium from below, and thus are a more structurally and biologically accurate representation of the human respiratory microenvironment. Attempts to repurpose existing drugs to SARS-CoV-2 treatment identified the anti-malaria drug chloroquine, which demonstrated potent antiviral activity against SARS-CoV-2 in a submerged cancer cell culture model (Vero E6 cells) (4). However, it was later proved to have limited efficacy in clinical trials and was confirmed to be unsuccessful in reducing SARS-CoV-2 infection in ALI tissue culture models (5–7). Nirmatrelvir and remdesivir were weak but showed good efficacy in ALI-culture as well as clinical trials (8, 9), perhaps indicating that these ALI models more accurately simulate human airway tissue responses. In fact, the kinetics of virus replication seen in ALI-culture was similar to human virus challenge or clinical studies. For example, viral peak and the level of nasal virus shedding seen in ALI-culture was similar to viral kinetics observed in SARS-CoV-2 or RSV human challenge studies (10–13). Thus, RSV is known to replicate well in this *in vitro* model and the infection system have been well established as previously published (12, 14–16).

Thus, it indicates that these ALI models more accurately simulate human airway tissue responses. In addition, ALI-culture, therefore, contributes to the 3Rs strategy (Replacement, Reduction and Refinement) of animal experiments. Furthermore, this is a useful model to evaluate new types of inhaled or intranasal antiviral agents *via* treatment of the exposed apical surface (17).

A major downside of the use of ALI-culture cells is the difference in responses to viruses by different batches of cells. As the primary cells are obtained from different donors to prepare ALI epithelium, this is an obvious and well acknowledged outcome. Consequently, donors from different diseases such as asthma, cystic fibrosis and chronic obstructive pulmonary disease, or having smoking history are usually carefully selected and studied separately. However, other factors such as age and gender are often ignored, or occasionally epithelial cells from only one donor were used as a representative for research or publication. Elderly people are known to be vulnerable to virus infection (18). However, the impact of cellular senescence on virus infection is controversial (19, 20). This has not been appropriately tested in the *in vitro* setting due to lack of proper virus infection models. At least, virus infection was reported to induce cellular senescence (21, 22), but this is not proof that virus is susceptible to senescent cells. Vom Steeg and Kelin (23) have described that, during viral infections, females have greater inflammatory, antiviral, and humoral immune responses compared with males, although males are more susceptible to virus infection, but again this has not been considered when conducting respiratory virus infection studies using ALI-culture epithelium.

Human RSV is a single-stranded, negative-sense RNA virus and a member of the family of Pneumoviridae of the Mononegavirales order. RSV infection is increasingly implicated as a cause of exacerbations in patients suffering from chronic obstructive pulmonary disease (COPD) (24), asthma (25) and cystic fibrosis (26). In immuno-compromised adults, approximately 50% of upper respiratory tract infections with RSV progress to pneumonia. In addition, RSV infection is the most common cause of childhood acute lower respiratory infection (27), and can produce severe disease in patients of any age. Even more importantly, the elderly population are reported to be particularly vulnerable (28), which is the reason why we selected RSV for the current study. Therefore, the aim of this report is to investigate the impact of age on RSV infection in pseudostratified ALI-culture bronchial epithelium.

## Materials and methods

### Cells and virus

Human larynx epithelial (HEp-2) cells (HeLa cell contaminant) (ATCC® CCL-23™) were purchased from the American Tissue Culture Collection (ATCC, Manassas, VA, United States) and maintained in 10% foetal bovine serum (FBS) supplemented DMEM with phenol red (# 4190–094: Life Technologies Ltd., Paisley, United Kingdom) at 37°C/5% CO_2_. MucilAir™ bronchial epithelium was provided fully differentiated as 24-well plate sized inserts by Epithelix Sàrl (Geneva, Switzerland). Cells were isolated from the lung tissue without any characteristics of cancer cells based on pathological analysis (Supplementary Table S1). Twice weekly, MucilAir™ inserts were transferred to a new 24-well plate containing 780 μL of MucilAir™ culture medium (EP04MM), and the apical surface was washed weekly with 400 μL PBS (once). MucilAir™ cultures were incubated at 37°C, 5% CO_2_. RSV A2 strain was obtained from the National Collection of Pathogenic Viruses (Public Health England) and passaged in HEp-2 cells containing DMEM supplemented with 2% (v/v) FBS to generate a virus stock solution (1.3 ~ 1.7 × 10^5^ PFU/mL). Fifty percent (w/v) sucrose in PBS was added to clarified culture supernatants to a final volume of 12.5% (v/v) sucrose solution.

### Infection and treatment of MucilAir™ culture

Prior to infection, MucilAir™ cultures were washed once with PBS and transferred to a new 24-well plate containing MucilAir™ culture medium. Virus was inoculated by adding 2000 PFU (an approximate multiplicity of infection of 0.01) of RSV stock solution to the apical surface of each well for 1 h. We previously tested with inoculum at 0.00001 to 1 MOI and found 0.01 is the minimum inoculum to achieve high peak viral load without acute cell toxicity (data not shown). Virus inoculum was then removed, and the apical surface washed twice with PBS. A third apical wash using 300 μL of PBS was collected and added to 100 μL PBS containing 50% (w/v) sucrose to generate a baseline (Day 0) for viral load and cytokine assessment. On subsequent days (Day 1–10), 300 μL of PBS was applied to the apical surface, and this wash was collected daily for viral load and cytokine assessment. On Day 5 post inoculation, the basolateral medium was removed from all wells and replenished with fresh MucilAir™ culture medium as a necessary maintenance step for MucilAir™ inserts.

### Determination of viral load by plaque assay

HEp-2 cells were seeded into 24-well plates (Corning, NY) at a density of 5 ~ 10 × 10^4^ cells/well and grown for 48-h prior to infection in 10% FBS DMEM until they attained 100% confluency. Collected apical wash samples were thawed at room temperature and 10-fold serial dilutions were prepared in serum-free DMEM. The growth medium from HEp-2 cells was aspirated and replaced with 300 μL of serially diluted virus collections and left to infect at 37°C/5% CO_2_ for 4 h. The infectious media was aspirated and replaced with 1 mL of Plaque Assay Overlay [0.3% Avicel RC-591 (FMC Biopolymer United Kingdom, Girvan, Scotland)] in MEM, supplemented to a final concentration of 2% FBS), and incubated for 7 days at 37°C/5% CO_2_. Cells were fixed with ice-cold methanol for 10 min before methanol was removed and cells washed twice with sterile PBS. Cells were then stained with 200 μL of 0.1% crystal violet solution (in distilled water) for 1 h. Crystal violet solution was removed, and cells were rinsed with water before plaques were counted and viral load enumerated.

### Viral RNA extraction and quantitative reverse transcriptase polymerase chain reaction for respiratory syncytial virus a nucleoprotein

Viral RNA was extracted from collected samples and the RSV A2 inoculation stock solution using a MagMAX™-96 Viral RNA isolation kit (Ambion by Life technologies) as per the manufacturer’s instructions before being subjected to quantitative PCR analysis using the One-Step qRT-PCR system (Primer Design, Southampton, United Kingdom). Briefly, 5 μL of extracted viral RNA was mixed with 10 μL OneStep qRT-PCR master mix, 4 μL RNase/DNase free water and 1 μL of the RSV A primer/probe mix (Cat # Path-RSV-A-standard, Primer Design, Southampton, United Kingdom) per reaction, with reactions being performed in duplicate. PCR plates were sealed with MicroAmp™ optical adhesive film (Cat # 4311971, life technologies) and briefly centrifuged at 1200 RPM. The One-Step PCR reaction and subsequent amplification analysis was carried out using an Applied Biosystems StepOnePlus™ Real-Time PCR System (Cat # 4376598, Life Technologies) using the following condition; 55°C for 10 min and 95°C for 8 min, followed by 50 cycles of qPCR at 10 s at 95°C and 60 s at 60°C. Reactions containing 10-fold serial dilutions of RNA extracted from the stock RSV A2 virus solution were used to generate a standard curve against which the RSV RNA content measured from test samples was quantified.

### Cytokine analysis

Collected apical washes were subjected to cytokine analysis using standard Ultra-Sensitive Meso Scale Diagnostics (MSD) assays for RANTES, V-Plex IL-8 MSD assays for IL-8, Human IP-10 Tissue Culture Kit for CXCL10, and Human proinflammatory 9-plex TC assay or Human IL-6 Tissue Culture Kit for IL-6 (all: MSD, Rockville, MA). Measurement of RANTES, CXCL10, and CXCL8/IL-6 required dilution of samples 1:2, 1:5 and 1:10, respectively, in Reagent Diluent. The electrochemiluminescence signal of serially-diluted standard samples, provided with each assay kit, was measured using a MESO QuickPlex plate reader and used to generate a standard curve using Discovery Workbench 4.0 software for each analyte. Each apical wash sample was quantified using these standard curves.

### Mucin quantification

Mucin concentrations were quantified using an enzyme-linked lectin assay (ELLA) based on the protocol previously described (29). Briefly, samples were sonicated for 10 min and added to high-bind ELISA plates coated in lectin from *Triculum vulgaris*. A standard curve was prepared using serially diluted mucin of known concentration from bovine submaxillary gland. Following incubation at 37°C for 30 min, the plates were washed three times with a wash buffer before the addition of detection reagent (containing HRP-conjugated Glycine max soybean lectin) for a further 30 min at 37°C. Following a further wash cycle, a substrate solution containing H_2_O_2_ and tetramethylbenzidine (R&D Systems, Minneapolis, MN) was added and allowed to develop for 5 min. The reaction was terminated using 2 M H_2_SO_4_, absorbance was read immediately at 450 nm with 570 nm as reference. Standard curves were created using GraphPad Prism and these were used to calculate the concentration of mucin in all samples.

### Double stranded DNA quantification

The concentration of dsDNA in apical washes was quantified using a Quant-iT PicoGreen dsDNA Assay Kit (Life technologies) as per manufacturer’s instructions. A standard curve was prepared by serially-dilution of a 2 μg/ml stock of dsDNA (Phage lambda DNA), and then incubated in the presence of the Quant-iT™ PicoGreen® dsDNA reagent for 3 min. Samples were added to the plates at a 1:2 dilution in TE buffer and incubated for 3 min in the presence of Quant-iT™ PicoGreen® dsDNA reagent, and the fluorescence of each well [545 nm (excitation) / 590 nm (emission)] was determined using a monochromator microplate reader (CLARIOstar®: BMG Labtech, Buckinghamshire, United Kingdom). A standard curve was created using MARS data analysis software (BMG Labtech, Buckinghamshire, United Kingdom) and the equation used to calculate concentration levels including the dilution factor used.

### Lactate dehydrogenase assay

LDH in apical washes was quantified using a CyQuant LDH Cytotoxicity assay kit (Thermo Fisher Scientific Inc. Basingstoke, United Kingdom) as per manufacturer’s instructions. Samples were added to the plates at a 1:2 dilution and incubated with Reaction Mixture for 30 min at room temperature, and the reaction was stopped by adding Stop Solution. The absorbance of each well at 490 nm and 680 nm was determined using a monochromator microplate reader (CLARIOstar®: BMG Labtech, Buckinghamshire, United Kingdom). The LDH activity was provided as OD, by subtracting the 680 nm OD from the 490 nm OD.

### p21/CDKN1A RT-qPCR

RNA extraction was performed using the RNeasy plant mini kit (Qiagen Ltd., Manchester, United Kingdom) in accordance with manufacturer’s instructions. RNA was stored at −80°C if not used immediately for RT-qPCR. TaqMan® RNA-to-CT ™ 1-Step Kit (Thermo Fisher Scientific, Basingstoke, United Kingdom) was used for gene expression analysis according to manufacturer’s instruction. Briefly, kit components and the primers pair (p21/CDKN1A:#Hs01121172_m1,GAPDH:#Hs99999905_m1, Thermo Fisher Scientific) were combined in a MicroAmp™ Fast Optical 96-Well Reaction Plate, 0.1 mL (10μLTaqMan® RT-PCR Mix (2x), 1 μL TaqMan® gene expression assay (20X), 0.5 μL TaqMan® RT Enzyme Mix (40x), 5 μL RNA template [up to 1 μg), 3.5 μL RNase-free H2O (up to Σ20μL)] was covered with MicroAmp optical adhesive film, briefly centrifuged. The PCR reaction was carried out using the Applied Biosystems StepOnePlus™ Real-Time PCR System with the run cycle (x1 reverse transcription, 30 min at 48°C, x1 cycle enzyme activation, 10 min at 95°C, x40 cycles (Denaturation 15 s at 95°C and Data collection, 1 min at 60°C). Fold change in gene expression vs. GAPDH was calculated as ΔCT.

### Statistical analysis

Results were represented as mean ± standard error of the mean or standard error as indicated. AUC (DAY 3–10) was calculated using GraphPad Prism (GraphPad Software, Inc., La Jolla, CA), and min-max with median was shown for grouping analysis. The comparison between two groups was performed by unpaired non-parametric Mann–Whitney test or *t*-test with Welch’s correction using GraphPad Prism. Correlation analysis between indicated parameters was also conducted by non-parametric Spearman test with statistical significance defined as *p* < 0.05.

## Results

### An impact of age on respiratory syncytial virus A2 viral load

RSV A2 was inoculated apically to the ALI-culture bronchial epithelium, and apical washed collected daily. Time-profiles of viral load (live virus by plaque assay, virus genome by RT-PCR) in apical washes were analyzed in donors from two different age groups, ≤60 years old donors (*n* = 4, 46.8 ± 13.7) and > 65 years old donors (*n* = 4, 68.5 ± 3.51; Table 1; Supplementary Table S1).

**Table 1:** Comparison of basal biomarker levels between younger (≤60 years old) and elderly (>65 years old) donors.

	≤60 years old donors	>65 years old donors	Statistical analysis
*n*	4	4	
Age	46.8 ± 13.7	68.5 ± 3.51	*p* < 0.05
Gender male/female	3/1	2/2	N/A
Basal CXL8 (pg/mL)	376 ± 507	73.1 ± 141	NS
Basal IL-6 (pg/mL)	15.4 ± 16.4	0.858 ± 0.495	NS
Basal RANTES (pg/mL)	1.01 ± 0.975	0.667 ± 0.996	NS
Basal CXCL10 (pg/mL)	88.2 ± 67.4	15.8 ± 18.7	NS
Basal mucin (AU)	348 ± 218	499 ± 474	NS
Basal dsDNA (ng/mL)	15.4 ± 81.2	43.6 ± 49.5	NS
Basal p21^CDKN1A^ (vs. GAPDH, RT-PCR)	0.313 ± 0.181	0.781 ± 0.220	*p* < 0.05

Mean ± SD is shown. Basal biomarker levels were determined in intact bronchial epithelium. PFU, plaque forming units. AU: absorbance units, NS: not significant.

**Figure 1:**
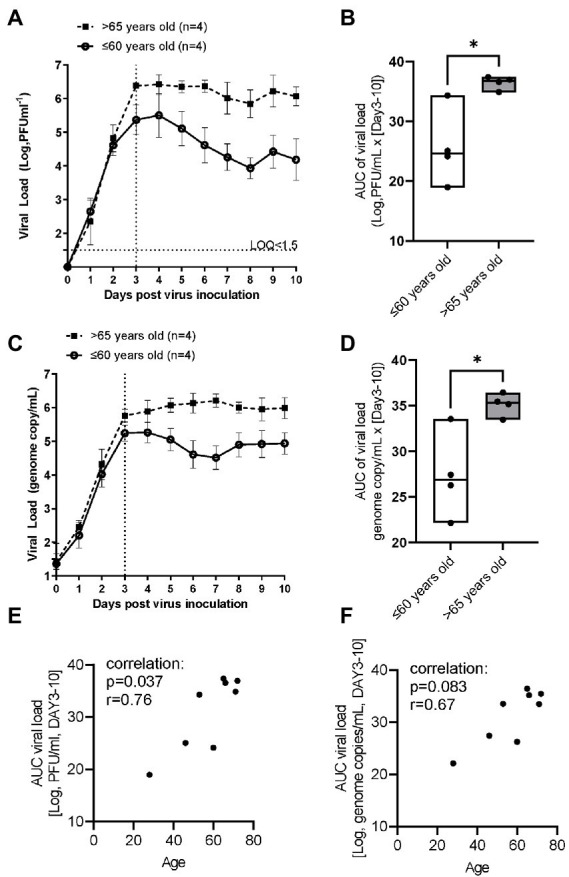
The comparison of RSV A2 viral load in apical washes collected from ALI-bronchial epithelium post RSV A2 inoculation in younger (≤60 years old) and elderly (>65 years old) groups. **(A)** Time-profile of viral load determined by plaque assay, mean ± SEM. **(B)** AUC (Day 3–10) of viral load determined by plaque assay, min-max with median. **(C)** Time-profile of viral load determined by qRT-PCR. **(D)** AUC (Day 3–10) of viral load determined by qRT-PCR. A correlation between age and viral load by plaque assay **(E)** and qRT-PCR **(F)**. *p < 0.05, the LOQ is 1.5 log, PFU/mL for a plaque assay, and virus load lower than LOQ was shown as <1.5.

In the younger group (≤60 years old), following inoculation with a low level of RSV A2 (0.01 MOI), virus replicated well, and the virus load (plaque assay) peaked on Day 3, and then gradually reduced up to Day 10 (Figure 1A). In contrast, in the elderly group (>65 years old), although the peak day and peak viral load were similar to those in younger group, RSV viral load did not reduce substantially up to Day 10 post inoculation, suggesting impaired virus clearance (Figure 1A; Table 2). This was also proven as significantly lower slope of viral load reduction in elderly group compared with that of younger group (Table 2). Consequently, viral load AUC in the elderly group was significantly higher than that in younger group (Figure 1B). There was no correlation between the peak viral load and virus clearance (either slope of virus reduction at Day 3–6 or at Day 3–10), [Spearman r = −0.60 *p* = 0.13 and r = −0.59 p = 0.13, respectively], and therefore, the peak viral load will not be a driving factor for virus clearance. This was also confirmed by the viral load determined as the genome copies of RSV by RT-PCR (Figures 1C,D). In addition, there was a statistically significant and a trend of positive correlation between age and viral load determined by plaque assay or and RT-PCR, respectively (Figures 1E,F).

**Table 2:** Comparison of viral infection parameters and biomarker levels between younger (≤60 years old) and elderly (>65 years old) donors.

	≤60 years old donors	>65 years old donors	Statistical analysis
*n*	4	4	
Viral peak (day)	3.75 ± 0.500	3.50 ± 0.577	NS
Viral peak (Log, PFU/mL)	5.63 ± 1.08	6.50 ± 0.197	NS
Viral load (slope Day 3–6)	0.251 ± 0.116	0.005 ± 0.061	*p* < 0.05
Viral load (slope Day 3–10)	0.169 ± 0.107	0.0446 ± 0.0450	NS
Viral load (AUC [log, PFU/mL] Day 3–10)	25.6 ± 6.38	36.5 ± 1.09	*p* < 0.05
PCR Viral load (AUC [log, copy/mL] Day 3–10)	27.3 ± 4.70	35.1 ± 1.23	*p* < 0.05
AUC CXCL8 (pg/mL) Day 3–10	7,590 ± 3,440	13,000 ± 6,350	NS
AUC IL-6 (pg/mL) Day 3–10	246 ± 266	137 ± 160	NS
AUC RANTES (pg/mL) Day 3–10	87.2 ± 41.0	622 ± 416	*p* < 0.05
AUC CXCL10 (pg/mL) Day 3–10*	6,020 ± 2,670	133,000 ± 191,000	NS
AUC mucin (AU) Day 3–10	8,660 ± 6,580	156,000 ± 201,000	NS
AUC dsDNA (ng/mL) Day 3–10	1,920 ± 1,610	5,650 ± 1,280	*p* < 0.05
AUC LDH (OD) Day 3–10*	4.54 ± 0.540	5.61 ± 0.408	*p* < 0.05

Mean ± SD is shown. PFU, plaque forming unit, *n = 3 of ≤ 60 years old donors. AU: absorbance units, OD: optical density, NS: not significant.

### An impact of age on respiratory syncytial virus A2 induced cytokine production

**Figure 2:**
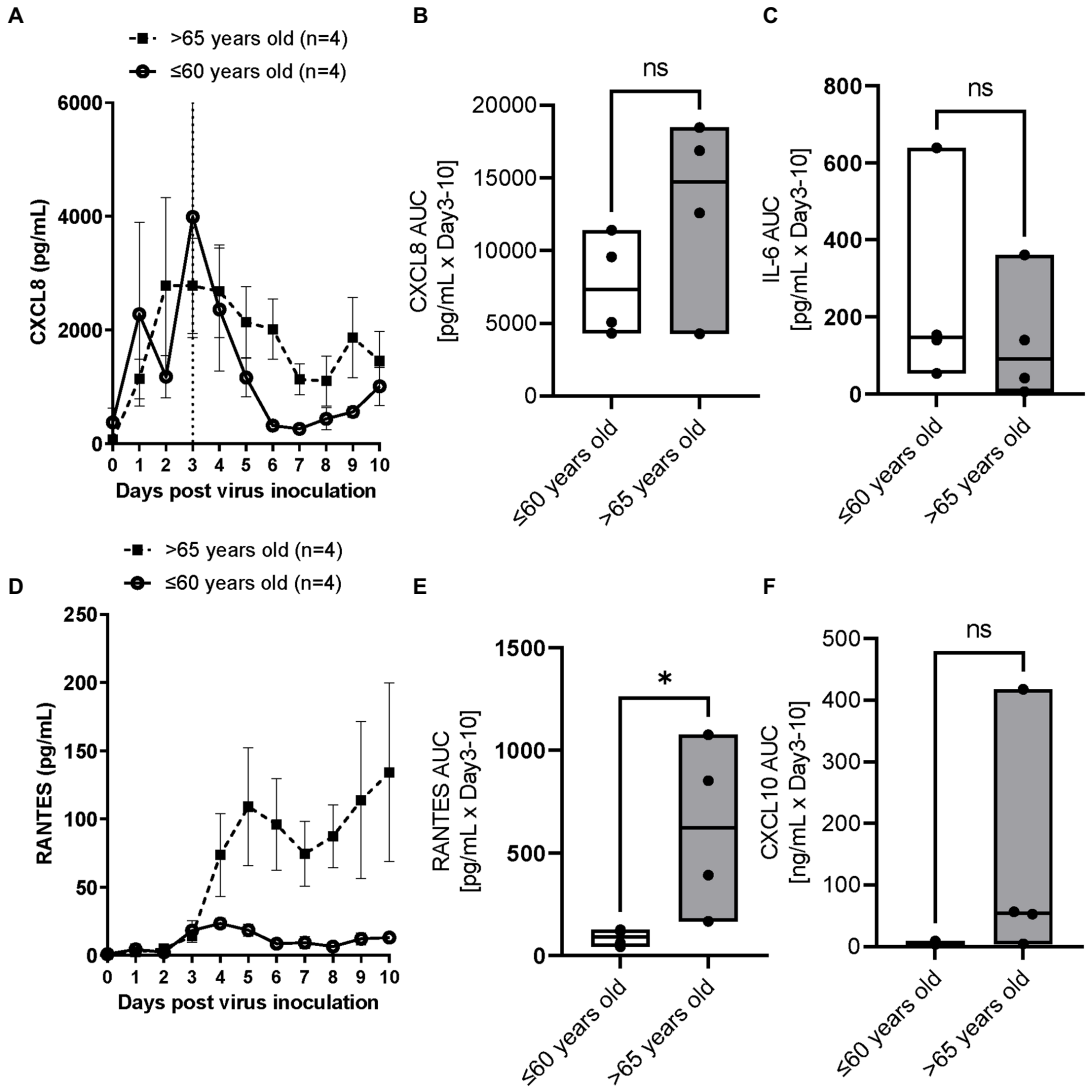
The comparison of RSV A2 induced cytokine biomarkers in apical washes collected from ALI-bronchial epithelium post RSV A2 inoculation in younger (≤60 years old) and elderly (>65 years old) groups. **(A)** Time-profile of CXCL8, **(B)** AUC (Day 3–10) of CXCL8, **(C)** AUC (Day 3–10) of IL-6, **(D)** Time-profile of RANTES, **(E)** AUC (Day 3–10) of RANTES, **(F)** AUC (Day 3–10) of CXCL10, mean ± SEM or min-max with median *p < 0.05. NS: not significant.

RSV A2 replication stimulated CXCL8 production in apical washes, which peaked on Day 3 post virus inoculation and then reduced over times, in parallel with viral load shown in Figure 1A in the younger group (Figure 2A). In contrast, the reduction rate of CXCL8 after peak was lower in the elderly group (Figure 2A), and AUC of CXCL8 (Day 3–10) trended towards being higher in elderly group than in younger group (Figure 2B; Table 2). There was a trend of positive correlation between CXCL8 and age, but not statistically significant (Supplementary Table S2). We did not observe any significant difference in basal CXCL8 levels (Table 1). RSV A2 replication also stimulated IL-6 production in apical washes, but the AUC of IL-6 was slightly higher in younger group although it was not statistically significant (Figure 2C; Table 2; Supplementary Figure S1A).

RSV A2 replication strongly and continuously stimulated RANTES production in apical washes, from Day 3 post virus inoculation, when virus load peaked, in the elderly group although there was limited induction in RANTES in the younger group (Figure 2D). Consequently, the AUC of RANTES was significantly higher in the elderly group than the younger group (Figure 2E; Table 2). We also observed a similar trend in virus induced CXCL10 induction (Figure 2F; Table 2; Supplementary Figure S1B).

### An impact of age on respiratory syncytial virus A2 induced mucin, dsDNA and LDH release

**Figure 3:**
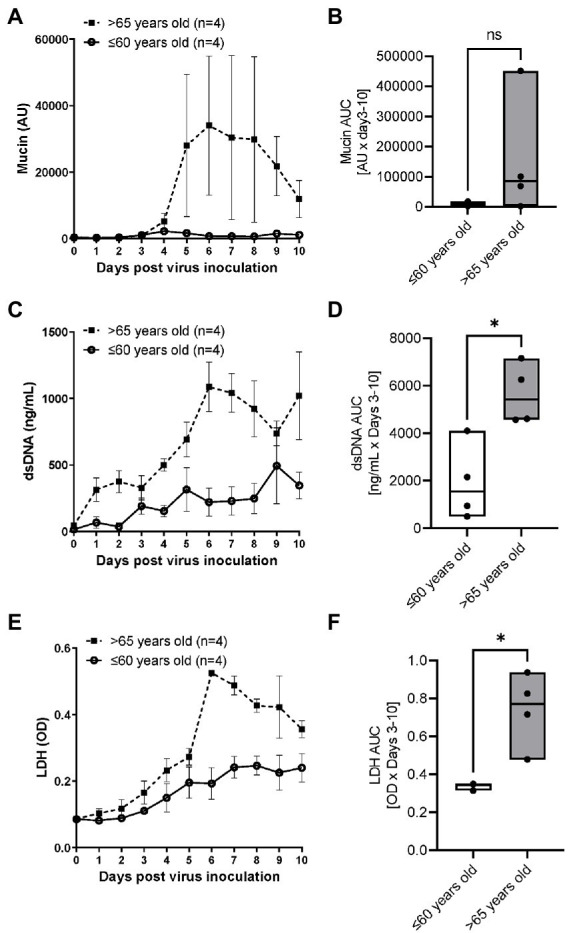
The comparison of RSV A2 induced mucin production and dsDNA/LDH release in apical washes collected from ALI-bronchial epithelium post RSV A2 inoculation in younger (≤60 years old) and elderly (>65 years old) groups. **(A)** Time-profile of mucin production, **(B)** AUC (Day 3–10) of mucin, **(C)** Time-profile of dsDNA, **(D)** AUC (Day 3–10) of dsDNA. **(E)** Time-profile of LDH, and **(F)** AUC (Day 3–10) of LDH. Mean ± SEM, *p < 0.05. AU: absorbance units, OD: optical density, NS: not significant.

RSV A2 replication stimulated mucin production in apical washes after Day 4 post virus inoculation (Figure 3A) and the AUC trended to being higher in the elderly group than the younger group (Figure 3B). dsDNA, as a marker of cell damage at the apical site, was also increased after virus inoculation, peaked at Day 6 post virus inoculation in elderly group, but the dsDNA release was less in the younger group, which was confirmed by significantly higher AUC of dsDNA in the elderly group (Figures 3C,D; Table 2). There was no difference of baseline mucin and dsDNA between the elderly and younger groups (Table 1). LDH, as another marker of cell damage at the apical site, was also increased after virus inoculation, peaked at Day 6 post virus inoculation in elderly group, but the LDH release was less in the younger group, which was confirmed by significantly higher AUC of dsDNA in the elderly group (Figures 3E,F; Table 2). There were statistically significant positive correlations of dsDNA or LDH with age (Supplementary Table S2).

### Basal p21 gene expression

**Figure 4:**
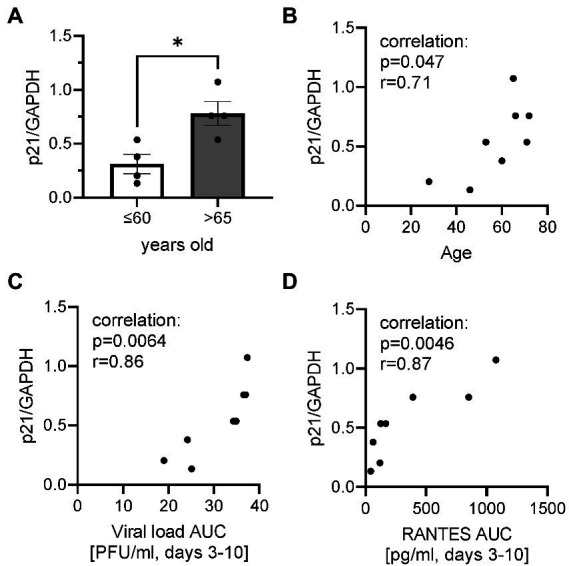
Basal p21 gene expression normalized by housekeeping gene GAPDH. **(A)** The comparison of basal p21 gene expression in cells collected from ALI-bronchial epithelium in younger (≤60 years old) and elderly (>65 years old) groups. A correlation between p21 gene expression and age **(B)**, plaque assay-viral load (Log, PFU/ml) AUC **(C),** or RANTES AUC **(D)**. *p < 0.05.

To investigate whether cells were senescent before infection, the basal level of gene expression of p21^CDKNA1^ was determined by RT-PCR in epithelium (non-infection) as a cellular senescent marker. The p21 gene expression was significantly higher in elderly group (Figure 4A). There were significant positive correlations between p21 gene expression and age, viral load AUC and RANTES AUC (Figures 4B–D).

## Discussion

In this paper, we demonstrated for the first time that airway epithelium obtained from an older population showed an impaired virus clearance with increased chemokines, cell damage and mucin production, which were correlated with the level of cellular senescence before infection. Although respiratory viruses are reported to induce cellular senescence in airway epithelial cells upon infection, this is important findings that RSV A2 was susceptible to aged cells, providing a potential reason why older populations are vulnerable to respiratory virus infections.

Toapanta and Ross (30) demonstrated that older mice showed impaired virus clearance due to delayed immune response, however, in contrast, several reports showed that aging was protective to virus infection (31, 32). For aging research, 18 month old mice are usually used, which is roughly equivalent to the end of human middle age or the beginning of old age, according to the information provided by the Jackson Laboratory (33). In humans, generally 65 years is used as the fixed threshold at which old age and economic dependency begins based on the old social security act, which might be redefined as the life expectancy becomes longer in current times. Thus, aged mouse studies might not translate to the events in elderly people. In fact, in this study, although we had limited donors, analysis revealed that a threshold of 65 years is still the best to demonstrate differences between elderly and younger donors compared with thresholds of 55 and 70 years old (data not shown). However, the fact remains that the low sample number severely limits the meaningful interpretation of the data regarding the age threshold, and further studies should be necessary. It should be noted that patients are usually admitted to hospital after being symptomatic, meaning after virus infection is well established or after peak, therefore, an accurate time-course cannot be measured. In this study, we did not find much difference in peak viral load or peak day post inoculation, however, clear differences between groups were seen in clearance of virus at later timepoints. Apoptosis of virus infected cells is known to be one of the mechanisms for host cells to tackle virus infection (34). However, senescence cells are known to be anti-apoptotic (35), and therefore, virus could potentially continuously replicate in the senescent cells, thereby acting as a kind of reservoir for viruses. This has not been reported or confirmed yet, and further studies using senescent cells are required.

Thus, we found the viral clearance was impaired in elder population without any statistical difference in peak viral load (Figures 1A,B; Table 2). There was no correlation between the peak viral load and the virus clearance determined as the slope of virus reduction as described in Results section. Thus, the peak viral load will not be a driving factor of poor viral clearance. Further studies with different inoculum size will help to understand the difference in the future.

ALI-cultures were also characterized by increases in the concentrations of several biomarkers. RANTES has been shown to correlate with RSV disease severity (36) and with RSV load in humans (37). CXCL8 and IL-6 were also reported to increase in nasal aspirate after infection and show a strong correlation with symptoms (36). RSV is known to be a strong mucus producer. In this report, we also found an increase in CXCL8, IL-6, RANTES, CXCL10, mucin, LDH as well as dsDNA, a potential marker of cell damage. Interestingly, RANTES, dsDNA and LDH release were significantly (but mucin, CXCL10 and CXCL8 not significantly) higher in the elderly population. However, IL-6 tended to be higher in younger and female populations (Table 2; Supplementary Table S3). Despite serving as a high-fidelity model of the human airway and observation of the difference between ages, there were some limitations that hindered data interpretation in this study. Firstly, the study was not appropriately powered to detect differences in some biomarkers. Although we observed significant difference on viral load, RANTES and dsDNA, more donors were required to demonstrate differences in CXCL8, mucin and others. In our previous studies, power calculations indicated that *n* = 3 per group is required for viral load but a larger sample size is needed to compare biomarkers due to greater variation between donors (12). In fact, we were able to show significant difference in viral load AUC between younger and elderly groups in this study with the sample size of *n* = 4 per group. Surprisingly, we also showed statistical differences in RANTES, dsDNA and LDH in limited sample size, but we need to increase donor number in future to obtain conclusive data. In addition, we observed cells from a 28-year-old donor showed the lowest viral load (peak and AUC) as well as lowest dsDNA level (cell damage) and lower levels in other biomarkers (data not shown). Therefore, it would be beneficial to divide the <60 years group into two groups of younger donors (20–40 years old) and middle age group (40–60 years old) to further investigate this. Secondly, gender balance of samples was not often considered when selecting donors of ALI epithelium. Vom Steeg and Kelin (23) have described that, during viral infections, females have greater inflammatory, antiviral, and humoral immune responses compared with males, although males are more susceptible to virus infection. For example, in an influenza challenge test, females were more likely to be symptomatic (38), and also Robinson et al. demonstrated that females suffer worse outcomes from influenza A infection than males (39). Conversely, a male bias in COVID19 mortality and inflammation was reported (40). In this study, the viral load and biomarkers AUC were compared between male and female donors (Supplementary Table S3) and no significant differences in peak day, peak viral load, slope of virus reduction after peak, CXCL8, RANTES and dsDNA were observed. IL-6 showed a trend of higher levels in females, and mucin production appeared to be higher in males, but these were not statistically significant. Thus, an impact of gender on RSV infection in ALI epithelium was inconclusive in this study due to lack of power. More studies are required, but the gender balance should be considered when conducting respiratory virus infection studies using ALI-culture epithelium. Thirdly, we measured gene expression of p21 (cellular senescence markers) in cell lysate of whole epithelium sheet at baseline (before infection) to understand the senescent level of cells before infection. Although we observed a good positive correlation between basal p21 expression and viral load, it is not clear whether the virus replicated more in senescent cells or not. As we do not believe that all cells were senescent, cell specific observations using imaging with virus and cellular senescent markers would help to understand this. In addition, the level of cellular senescence should be confirmed by other senescent markers such asp16, phosphorylated p53 and 402 Lamin B1. Fourthly, unlike these cultures, in the whole body, adaptive immune cells participate in virus elimination. In fact, although the peak virus load was similar to that observed in nasal wash samples collected from healthy subjects challenged with RSV Memphis 37 strain (41) or from RSV-infected infants (42), the viral load was sustained for more than 10 days in ALI epithelium whereas the virus infection resolved in 10 days in healthy subjects *in vivo*. Particularly, the vulnerability of aged population to respiratory virus infection is known to be caused by immune-senescence (43). Therefore, to obtain a more accurate picture, we need a co-culture system with immune cells from an aged population. Finally, ALI-culture itself still has some limitations. It is limited by the absence of a nutrient and waste transport system as well as by a lack of certain biomechanical pressures (to more accurately simulate breathing). However, a new pre-clinical model which takes into account both of these aforementioned limitations whilst maintaining the advantageous features of ALI-cultures, is coming to the fore of respiratory research; the airway-on-chip. This model consists of an advanced microfluidic cell culture device that also simulates *in vivo* vascular perfusion and biomechanical forces implicated in breathing, to provide the most accurate *in vitro* representation of the respiratory microenvironment to date (44). The system still has some problems such as lack of standardized protocols as well as its high cost and complexity to operate, but it will help to overcome the problems of ALI-culture for studying virus infections in the future.

In summary, we found aging was an important factor affecting respiratory virus infection and its clearance as well as virus associated biomarkers. Novel or innovative *in vitro* cell models are vital for researching respiratory virus infection or virus-associated exacerbations in chronic inflammatory pulmonary diseases, but as in clinical studies, the age (and potentially gender) balance of cell donors should be considered to obtain more translational results.

## Data availability statement

The raw data supporting the conclusions of this article will be made available by the authors, without undue reservation.

## Author contributions

KI conceived and designed the experiments. LD, MC, and KI performed the experiments and analyzed and interpreted the data. All authors contributed to the article and approved the submitted version.

## Funding

This study was partially funded by Pulmocide Ltd.

## Conflict of interest

KI was a co-founder and shareholder of Pulmocide Ltd., and currently serves as a consultant. MC was an employee and shareholder of Pulmocide Ltd. LD was an employee of Pulmocide Ltd.

## Publisher’s note

All claims expressed in this article are solely those of the authors and do not necessarily represent those of their affiliated organizations, or those of the publisher, the editors and the reviewers. Any product that may be evaluated in this article, or claim that may be made by its manufacturer, is not guaranteed or endorsed by the publisher.

## Abbreviations

ALI: air liquid interface
PFU: plaque forming unit
RSV: respiratory syncytial virus
LOQ: limit of quantification
