# Supplementary material for: An impact of age on respiratory syncytial virus infection in air-liquid-interface culture bronchial epithelium
Source: Front Med (Lausanne). 2023 Mar 14;10:1144050. doi: 10.3389/fmed.2023.1144050 (PMC10043235; doi:10.3389/fmed.2023.1144050)
Supplement: Supplementary file 1 [file Data_Sheet_1.pdf]

Supplementary Materials (Table and Figure)

Table S1 Donors of bronchial cells

Table S2 Correlation between age and biomarkers (AUC)

Table S3 Comparison of viral infection parameters and biomarker AUC between male and female donors

Figure S1 Time-profiles of IL-6 (A) and CXCL10 (B) release in apical wash post RSV inoculation in younger (≤60 years old) and elderly (>65 years old) groups.

Supplementary Table S1 Donors of bronchial cells

Donor No.	Gender	Age	Smoking history	COPD/asthma history	Pathology
1	Male	28	None	None	-ve
2	Female	46	None	None	-ve
3	Male	53	None	None	-ve
4	Male	60	None	None	-ve
5	Male	65	None	None	-ve
6	Female	66	None	None	-ve
7	Female	71	None	None	-ve
8	Male	72	None	None	-ve

Supplementary Table S2   Correlation between age and biomarkers (AUC)

(vs. Age)	Spearman r	p value
Viral peak (Log, PFU/mL)	0.45	0.27
Viral load (slope Day3-6)	-0.67	0.083
Viral load (slope Day3-10)	-0.68	0.10
Viral load (AUC [log, PFU/ml] Day3-10)	0.76	0.037*
PCR Viral load (AUC [log, copy/ml] Day3-10)	0.67	0.083
AUC CXCL8 (pg/mL) Day 3-10	0.31	0.46
AUC IL-6 (pg/mL) Day 3-10	-0.31	0.46
AUC RANTES (pg/mL) Day 3-10	0.71	0.058
AUC CXCL10 (pg/mL) Day 3-10 *	0.39	0.40
AUC mucin (AU) Day 3-10	0.43	0.30
AUC dsDNA (ng/mL) Day 3-10	0.79	0.028*
AUC LDH (OD) Day 3-10 *	0.79	0.048*

*n=7 (n=8 for all others)

Supplementary Table S3 Comparison of viral infection parameters and biomarker AUC between male and female donors

	male	female	Statistical analysis
n	5	3	
Age	55.6 ± 16.9	61.0 ± 13.2	NS
≤60 / >65 (years old)	3/2	1/2	N/A
Viral peak (day)	3.80 ± 0.447	3.33 ± 0.577	NS
Viral peak (Log, PFU/mL)	5.97 ± 1.11	6.22 ± 0.251	NS
Viral load (slope Day3-6)	0.120 ± 0.141	0.141 ± 0.214	NS
Viral load (slope Day3-10)	0.0929 ± 0.0954	0.130 ± 0.127	NS
AUC CXCL8 (pg/mL) Day 3-10	10,000± 5,300	10,800 ± 7,170	NS
AUC IL-6 (pg/mL) Day 3-10	105 ± 53.6	335 ± 317	NS
AUC RANTES (pg/mL) Day 3-10	30.4 ± 8.32	32.2 ± 6.21	NS
AUC mucin (AU) Day 3-10	109,000 ± 193,000	37,000 ± 55,200	NS
AUC dsDNA (ng/mL) Day 3-10	3,610 ± 2,090	4,090 ± 3,360	NS

Mean ± SD is shown. PFU: plaque forming unit.

Supplementary Figure S1

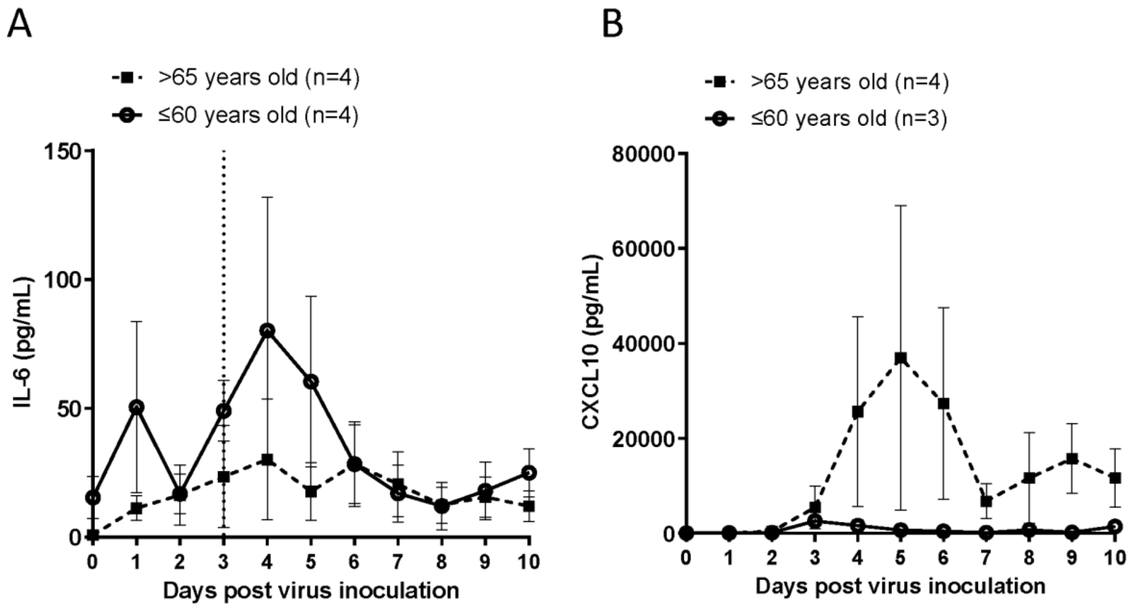

Time-profiles of IL-6 (A) and CXCL10 (B) release in apical wash post RSV inoculation in younger (≤60 years old) and elderly (>65 years old) groups. Mean ±SEM
